# Gut mycobiome dysbiosis after sepsis and trauma

**DOI:** 10.1186/s13054-023-04780-4

**Published:** 2024-01-11

**Authors:** Gwoncheol Park, Jennifer A. Munley, Lauren S. Kelly, Kolenkode B. Kannan, Robert T. Mankowski, Ashish Sharma, Gilbert Upchurch, Gemma Casadesus, Paramita Chakrabarty, Shannon M. Wallet, Robert Maile, Letitia E. Bible, Bo Wang, Lyle L. Moldawer, Alicia M. Mohr, Philip A. Efron, Ravinder Nagpal

**Affiliations:** 1 Department of Health, Nutrition, and Food Sciences, Florida State University, Tallahassee, FL 32306 USA; 2 Department of Surgery and Sepsis and Critical Illness Research Center, University of Florida College of Medicine, Gainesville, FL 32611 USA; 3 Department of Aging and Geriatric Research, University of Florida College of Medicine, Gainesville, FL 32611 USA; 4 Department of Pharmacology and Therapeutics, University of Florida College of Medicine, Gainesville, FL 32611 USA; 5 Department of Neuroscience, University of Florida College of Medicine, Gainesville, FL 32611 USA; 6 Department of Oral Biology, University of Florida College of Dentistry, Gainesville, FL 32611 USA; 7 Department of Biomedical and Chemical Engineering and Sciences, Florida Institute of Technology, Melbourne, FL 32901 USA

**Keywords:** Critical care, Critical illness, Dysbiosis, Fungi, Metabolome, Microbiota, Mycobiome, Pathobiome, Sepsis, Trauma

## Abstract

**Background:**

Sepsis and trauma are known to disrupt gut bacterial microbiome communities, but the impacts and perturbations in the fungal (mycobiome) community after severe infection or injury, particularly in patients experiencing chronic critical illness (CCI), remain unstudied.

**Methods:**

We assess persistence of the gut mycobiome perturbation (dysbiosis) in patients experiencing CCI following sepsis or trauma for up to two-to-three weeks after intensive care unit hospitalization.

**Results:**

We show that the dysbiotic mycobiome arrays shift toward a pathobiome state, which is more susceptible to infection, in CCI patients compared to age-matched healthy subjects. The fungal community in CCI patients is largely dominated by *Candida* spp; while, the commensal fungal species are depleted. Additionally, these myco-pathobiome arrays correlate with alterations in micro-ecological niche involving specific gut bacteria and gut-blood metabolites.

**Conclusions:**

The findings reveal the persistence of mycobiome dysbiosis in both sepsis and trauma settings, even up to two weeks post-sepsis and trauma, highlighting the need to assess and address the increased risk of fungal infections in CCI patients.

**Graphical Abstract:**

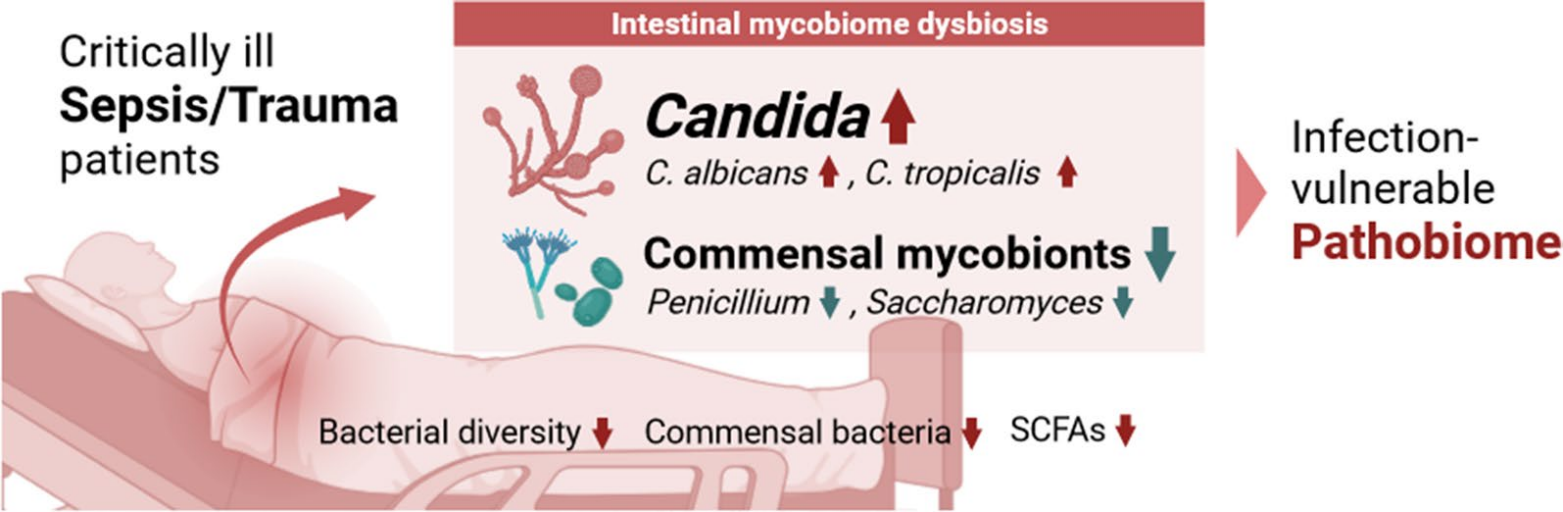

**Supplementary Information:**

The online version contains supplementary material available at 10.1186/s13054-023-04780-4.

## Background

The gut mycobiome (fungal microbiome) constitutes an important portion of the overall gut microbial community. While gut-associated fungi occupy a relatively smaller niche than bacteria, they play an important role in maintaining the gut bacterial microbiome, thus serving a crucial function in host homeostasis and physiological processes [1]. Over the last few decades, research has demonstrated that an imbalance in the gut mycobiome (‘dysbiosis’) could contribute to the pathogenesis of intestinal diseases, including inflammatory bowel disease [2, 3], irritable bowel syndrome [4, 5], and colorectal cancer [6]. Moreover, the consequences of a dysbiotic mycobiome are not restricted to the gastrointestinal tract and may in fact be closely associated with development of extra-intestinal diseases such as allergic airway disease by influencing local and peripheral immune homeostasis [7]. Indeed, excessive predominance of opportunistic pathogenic fungi (‘myco-pathobiome’) under dysbiotic conditions can lead to systemic fungal infections such as candidiasis that are potentially fatal [8]. Candidiasis is one of the most common causes of bloodstream infections in hospitalized patients [9, 10]. Due to the mutualistic ecological niche between bacteria and fungi, dysbiosis in bacterial communities can result in an imbalance in fungal communities and the overgrowth of pathogenic fungi [11]. Conversely, disruptions in fungal communities can also stimulate the growth of bacterial pathobionts, ultimately leading to the development or exacerbation of intestinal inflammatory diseases [12].

Sepsis and trauma represent two distinct medical conditions, with shared post-hospitalization clinical features and outcomes. Previous studies have shown that both sepsis and trauma patients, that are classified as ‘critically ill’, suffer extreme dysbiosis in the gut microbiome during hospitalization [13, 14], and this dysbiotic status closely correlates with the risk of in-hospital mortality [15, 16]. Gut dysbiosis in these critically ill patients may increase susceptibility to hospital-acquired infections, subsequent recurrence of sepsis, and multiorgan dysfunction syndrome [17]. Generally, critically ill patients may become immunocompromised or immunosuppressed due to the continuing medical conditions [18]. The compromised immune system results in uncontrolled proliferation of pathogens, allowing opportunistic pathogens and indigenous pathobionts to subvert the host immune system and alter the gut micro-ecological environment [19]. Importantly, antibiotics, commonly prescribed to sepsis and trauma patients to combat or prevent subsequent infections, destroy the beneficial gut commensals, triggering an infection-susceptible status [20].

We have previously observed persistent gut bacterial dysbiosis and altered metabolome profiles that shift toward a pathobiome state in critically ill patients 2–3 weeks after intensive care unit (ICU) admission. Given the strong and mutualistic relationship between bacterial and fungal communities, it is possible that that such expansive bacterial dysbiosis would lead to comorbid perturbations in the fungal microbiome community. However, there exists little to no information on the alterations in the broader fungal community, particularly in the later stages of sepsis and trauma patients who experience delayed recovery. Given that altered gut microbiome in these critically ill patients could underlie prolonged inflammation, immunosuppression, and catabolism (PICS) [21], we hypothesized that critically ill trauma and sepsis patients with delayed recovery will display a dysregulated mycobiome pattern. Our results show that the mycobiome profile in these critically ill patients shifts to a pathologic pattern dominated by *Candida* spp., with concurrent alterations in bacteriome-metabolome micro-ecological niches. Since the incidence of recidivism and the predisposition to poor long-term outcomes is high in these patients, our findings have direct implications and relevance for clinical considerations pertaining to the critical care and outcomes particularly in patients suffering from chronic critical illness, associated ICU hospitalization, and recovery therefrom.

## Methods

### Study population

**Fig. 1:**
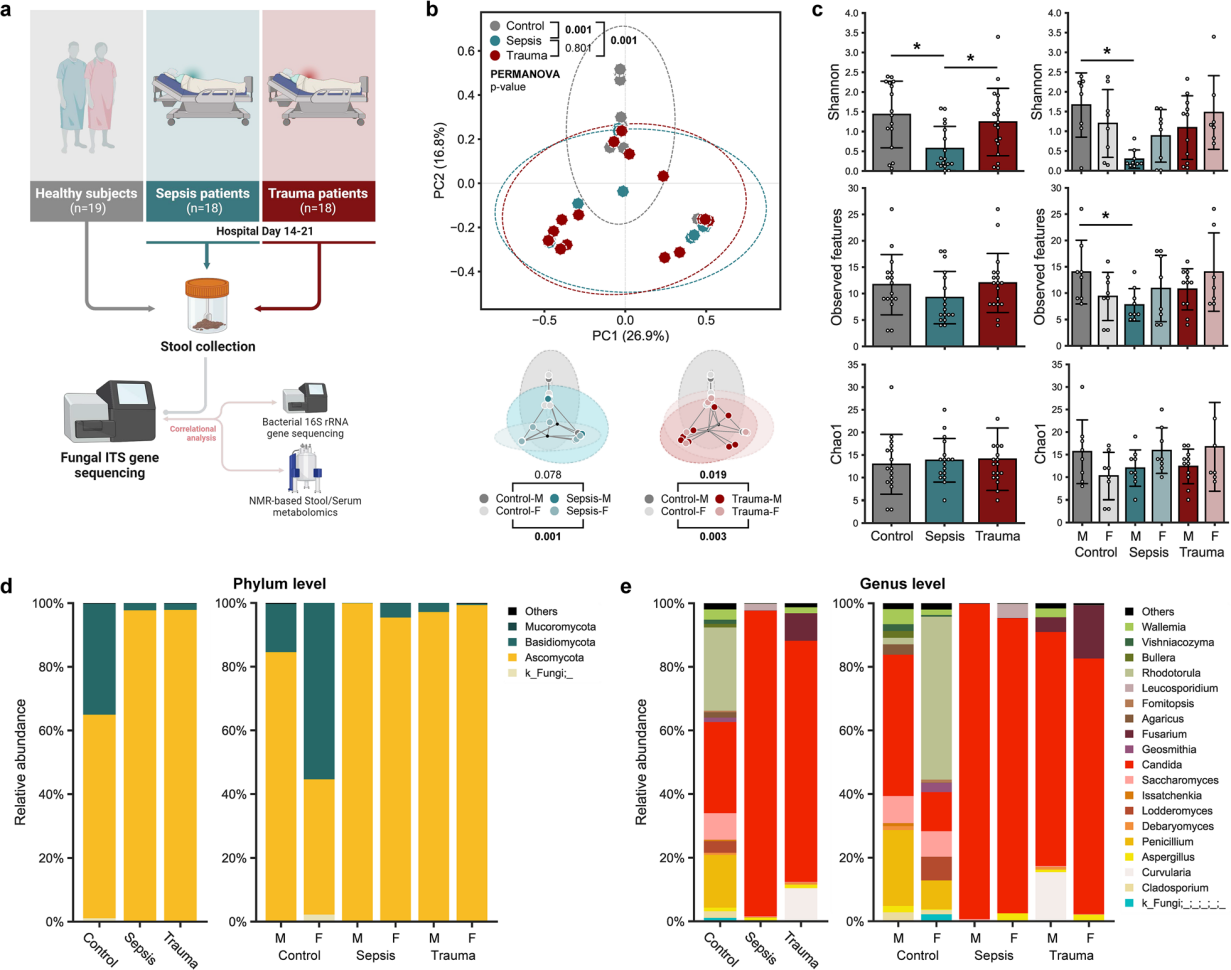
Sepsis/trauma patients have persistent fungal dysbiosis and pathobiome. **a** Schematic study design. **b** Principal coordinate analysis (PCoA) plots showing differences in beta-diversity (Bray–Curtis dissimilarity) between control, sepsis and trauma cohorts with PERMANOVA *p* values between cohorts. Beta-diversity differences between same sex-counterparts of sepsis or trauma versus control with associated *p* values. **c** Mycobiome alpha-diversity comparing control, sepsis and trauma cohorts represented by Shannon, observed amplicon sequence variants (ASVs), and Chao1 index and alpha-diversity comparing males between cohorts or females between cohorts. Intestinal microbiome microbial composition at the **d** phylum and **e** genus level between cohorts and separated by sex. M—males; F—females. Data are presented as mean ± SD. *p < 0.05

The overall experimental framework is depicted in Fig. 1A. This study was a single-center, prospective observational cohort investigation conducted from 2019 to 2023 at a Level-1 trauma center. The inclusion and exclusion criteria are detailed in Additional file 1: Table S1. Primary data collection for specific sepsis patients and healthy controls was carried out within the scope of the study titled “Persistent Inflammation, Immunosuppression, and Catabolism Syndrome (PICS): A New Horizon for Surgical Critical Care,” approved by the Institutional Review Board (IRB) under number 201400611 on October 28, 2014, and registered at ClinicalTrials.gov with the identifier NCT02276417. Initial data collection for certain trauma patients was conducted as part of the study titled “Hematopoietic Stem Cell Dysfunction in the Elderly after Severe Injury,” which received IRB approval under number 201601386 on September 6, 2016, and was registered at ClinicalTrials.gov with the identifier NCT02577731. The remaining data were gathered in the context of the study titled “Gut Microbiome Dysfunction in Sepsis and Trauma Survivors,” which received IRB approval under number 202102863 on May 3, 2022, and was registered at ClinicalTrials.gov with the identifier NCT05357170. This study’s reporting adheres to the guidelines outlined in the Strengthening the Reporting of Observational Studies in Epidemiology (STROBE) guidelines [22].

Ultimately, 19 healthy control subjects, 18 severely injured trauma patients, and 18 surgical sepsis patients were included. The selection of these sample sizes was based on a power analysis, which assumed an 80% or greater likelihood (beta) of detecting a 30% change from baseline, necessitating a group size of *n *= 18 for each group.

### Clinical data and sample collection

Clinical data, including age, gender, race, ethnicity, and medical comorbidities, were collected to profile the demographic features of the cohorts. Additionally, management and outcome parameters were documented, which included factors like the injury severity score, sepsis criteria scoring, blood product transfusions, antibiotic administration, dietary intake, as well as hospital and ICU lengths of stay. Mortality outcomes were also recorded. It is important to note that all trauma and sepsis patients included in the study underwent at least two weeks of standardized ICU management and met established criteria for Chronic Critical Illness (CCI) and PICS [23].

Stool specimens were acquired either per rectum, via a rectal tube, or from a colostomy between hospital days 14 and 21. These specimens were then divided into aliquots and preserved in vials containing Cary-Blair bacterial media (ThermoFisher Scientific, Waltham, MA) at -80ºC. Simultaneously, peripheral blood was obtained through a single venipuncture using heparinized blood collection tubes (Becton–Dickinson & Co., Franklin Lakes, NJ). The blood samples were centrifuged at 800 × g for 10 min, and the resulting plasma was preserved at − 80 °C.

### Mycobiome analysis

Mycobiome measurement and analyses were conducted as per our previously described method [24, 25]. Briefly, we extracted high-quality genomic DNA from 200 mg of fecal samples using the QIAmp PowerFecal Pro DNA Kit (Qiagen, Hilden, Germany) and quantified it with a Nanodrop spectrophotometer (Thermo Fisher Scientific, Waltham, MA, USA). The internal transcribed spacer (ITS) region of the fungal rRNA gene was amplified, following the Earth Microbiome Project benchmark protocol (www.earthmicrobiome.org). The resulting amplicons were purified using the AMPure^®^ XP magnetic beads kit (Beckman Coulter, Indianapolis, IN), and the purified products were quantified with the Qubit-4 fluorimeter (InVitrogen, Waltham, MA, USA). An amplicon library was generated following previously established methods [26]. The purified library was combined in equimolar concentrations and subjected to sequencing on an Illumina MiSeq platform (Illumina Inc., San Diego, CA, USA) using a 2 × 300 base pairs reagent kit (MiSeq reagent kit v3; Illumina Inc.) for paired-end sequencing.

### Bioinformatics and statistical analysis

Raw sequences were processed using the Quantitative Insights Into Microbial Ecology (QIIME2) bioinformatics software suite (version 2.2023.5) [27]. Quality-filtering, adapter-trimming, denoising, and removal of non-chimeric amplicons were conducted using the DADA2 pipeline through the q2-dada2-plugin with default parameters [28]. All identified amplicon sequence variants (ASVs) were aligned using MAFFT [29]. Taxonomic assignment for these ASVs was accomplished by employing the sklearn classifier, which utilized a pre-trained naïve Bayes taxonomy classifier. The classification process involved alignment against the 99% UNITE 9.0 database. Alpha-diversity was quantified using the number of observed ASVs, Chao1, and Shannon index metrics. To assess beta-diversity, the Bray–Curtis dissimilarity index was utilized, and the results were presented with a principal coordinate analysis (PcoA). Statistical analyses, including the nonparametric Kruskal–Wallis test and PERMANOVA with 999 random permutations, were applied to identify significant differences in microbial diversity and structure. The differential abundance of taxa was determined through the ANOVA-Like Differential Expression (ALDEx2) approach [30]. To predict the group based on microbial composition through supervised classification, the q2-sample-classifier plugin in QIIME2 was utilized. This process involved a nested stratified fivefold cross-validation using the Random Forest classifier, which was built with 5,000 trees. To evaluate the impact of two factors, group and sex, on taxa abundance and potential interaction effects, a two-way ANOVA was conducted. To examine the correlations between fungal taxa and bacterial taxa, gut, and plasma metabolites, Spearman’s rank correlation was used. Networks connecting fungal taxa and gut metabolites were established by calculating Spearman correlations, and significant associations (Spearman correlation coefficient > 0.30 and Benjamini–Hochberg corrected *p* value < 0.05) were visualized using Cytoscape v3.9.1 [31]. Data visualization was carried out using ‘R’ or ‘Python’ packages.

## Results

### Subject characteristics

Patient characteristics of healthy control subjects (control), sepsis patients, and trauma patients are summarized in Additional file 1: Table S2. No significant differences in age, sex, race, or ethnicity were observed. When comparing medical comorbidities (including hypertension, hyperlipidemia, renal disease with creatinine > 1.5 mg/dL, coronary artery disease, diabetes, congestive heart failure, chronic obstructive pulmonary disease, and cancer), significant distinctions among the groups were noted only for hypertension and coronary artery disease. Both the sepsis and trauma cohorts had a higher prevalence of hypertension compared to the control group, while the sepsis cohort exhibited a greater number of patients with a history of coronary artery disease. It is noteworthy that all sepsis and trauma patients received antibiotics during their hospital stay. As expected, sepsis patients received a larger variety of antibiotics for longer durations, primarily because all sepsis patients required antibiotics for source control. All patients in the sepsis and trauma groups received enteral nutrition, which included tube feeds or an oral diet. Only one sepsis patient received total parenteral nutrition for ten days. The sepsis group experienced significantly longer stays in the ICU and the hospital compared to the trauma cohort.

### Dysbiotic mycobiome signatures shift toward a pathobiome profile susceptible to infection following sepsis or trauma

**Fig. 2:**
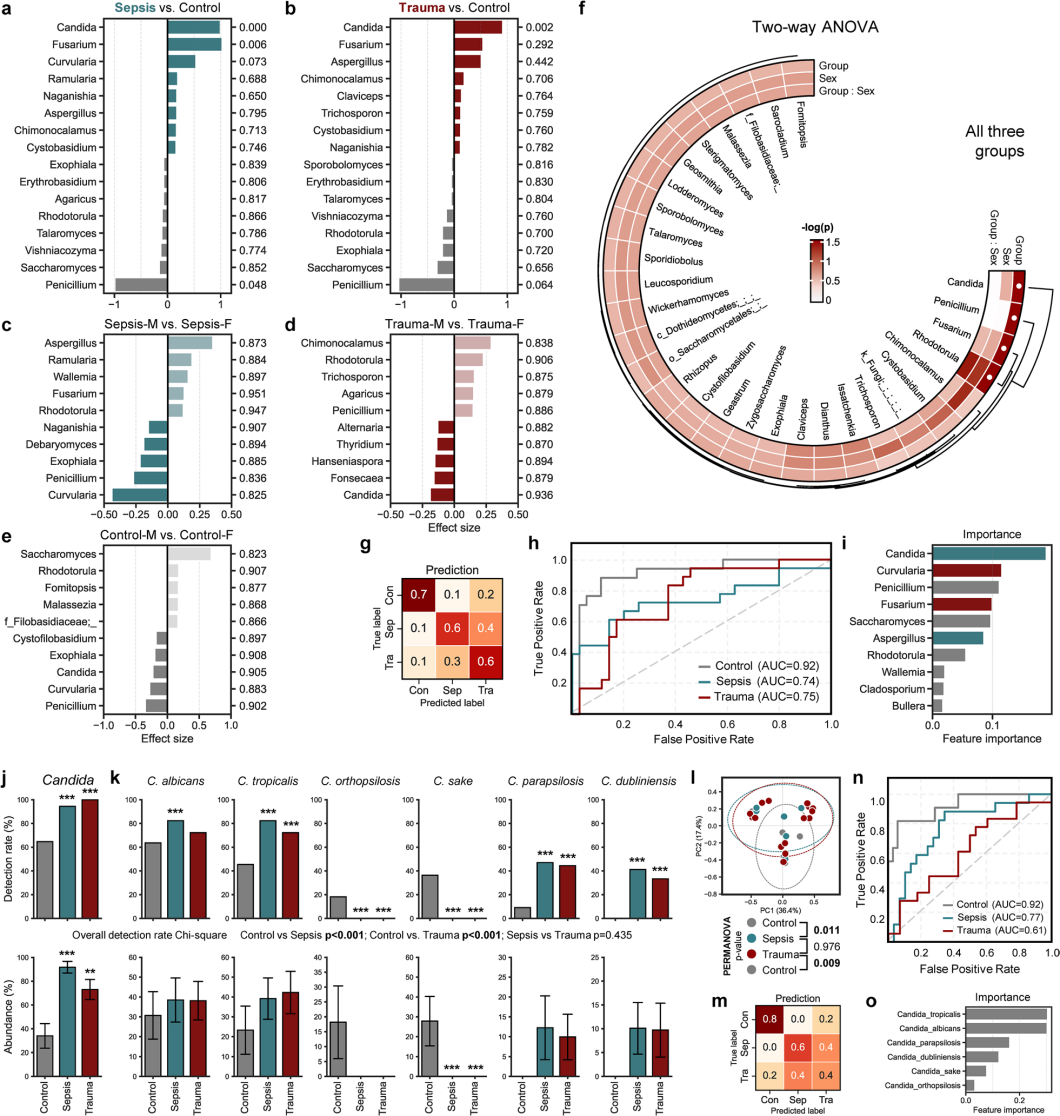
Dysbiotic mycobiome signatures in sepsis/trauma patients are primarily linked with pathobiont *Candida* species. Genera which are significantly more or less relatively abundant in either the **a** sepsis or the **b** trauma cohort compared to the control group, with *p* values displayed on the right column, and **c**–**e** differences between same sex-counterparts with associated *p* values. **f** Circular heatmaps showing two-way ANOVA results (group and sex) for all three groups comparing the mycobiota. *p* values were shown by converting to –log (Statistical significance: ● *p* value < 0.05). **g**–**i** A Random Forest prediction model between groups based on abundance data of mycobiome. **h** Receiver Operating Characteristic (ROC) curve depicts the classification accuracy; while, **i** a bar graph highlights the top 10 most strongly predictive genera based on relative importance scores. Detection rate and abundance of *Candida* **j** genus and **k** species. Difference in detection rate between groups was assessed using Chi-square test. The abundance of *Candida* species was determined by calculating the proportion (relative abundance) of each species within the overall *Candida* genus. **l** Principal coordinate analysis (PCoA) based on Bray–Curtis dissimilarity calculated with only the abundance of *Candida* species. **m**–**o** A Random Forest prediction model between groups based on abundance data of *Candida* species. **n** Receiver Operating Characteristic (ROC) curve depicts the classification accuracy; while, **o** a bar graph highlights the strongly predictive *Candida* species based on relative importance scores. **p < 0.01, ***p < 0.001

To investigate alterations in the gut mycobiome following hospitalization in critically ill sepsis and trauma patients, we conducted an evaluation of the fungal community diversity and composition. Additionally, we explored differences based on host sex (Fig. 1b, c). The overall mycobiome profiles of the sepsis and trauma cohorts were found to be nearly similar, while both significantly contrasted from the composition in the control cohort. In control group, the beta-diversity distance (a measure of the similarity of microbial communities between two samples or ecosystems) between subjects, transformed into a two-dimensional plot using PCoA analysis based on dissimilarity, exhibited closer proximity among females compared to males. This resulted in more distinctly divergent arrays between healthy adults versus sepsis or trauma patients among females (Fig. 1b), indicating the existence of dissimilar mycobiome profiles between these groups. In all groups, the microbial alpha-diversity (species richness) of patients exhibited a high degree of dispersion. However, sepsis patients displayed significantly lower microbial diversity compared to healthy adults and trauma patients, as measured by the Shannon index, which measures both microbial richness and evenness. While there were no differences between groups in the total number of amplicon sequence variants (ASVs, an indirect proxy for species number), which represent the observed count of genes potentially originating from different bacteria, and microbial diversity measured by the Chao1 index-measuring solely microbial richness-indicating no variation in microbial richness among the groups, only the sepsis group exhibited distinct microbial evenness. In the healthy control group, male subjects exhibited higher diversity than their female counterparts, whereas the opposite trend was observed in sepsis and trauma patients. Only male sepsis patients demonstrated significantly lower Shannon diversity and had fewer ASVs than healthy males. Male trauma patients also exhibited modestly reduced diversity than healthy males. In contrast, female sepsis and trauma patients demonstrated almost comparable or slightly higher diversity compared to healthy females (Fig. 1c). At the phylum level, the mycobiome community was dominated by two major fungal phyla, Basidiomycota and Ascomycota. It is noteworthy that Basidiomycota, which otherwise made up a considerable proportion of the composition in healthy adults, was nearly depleted in both sepsis and trauma patients (Fig. 1d). Further deeper analyses revealed that this difference was primarily due to an explosive increase in the levels of genus *Candida* in both sepsis and trauma patients. In healthy adults, *Rhodotorula*, *Saccharomyces*, *Penicillium*, and *Candida* were the major genera comprising the gut mycobiome, with *Candida* being the sole predominant genus in sepsis and trauma patients. Particularly in sepsis patients, *Candida* accounted for more than 95% of the mycobiome abundance on average (Fig. 1e). In differential abundance analysis, *Candida* was identified as the most significantly proliferated genus in both sepsis and trauma patients, followed by *Fusarium*, while *Penicillium* and *Saccharomyces* were nearly depleted (Fig. 2a, b). Additionally, further analysis using ANOVA distinguished *Candida*, *Penicillium*, *Fusarium*, and *Rhodotorula* as major taxa differing significantly among the three groups (Fig. 2f). Additionally, healthy adults exhibited distinct microbial compositions based on sex. Male adults had higher levels of *Candida* and *Penicillium*; while, female adults harbored more *Saccharomyces* and *Rhodotorula* (Fig. 1e, 2e). In both sexes, the abundance of taxa other than *Candida* was insignificantly low in sepsis and trauma patients, but *Aspergillus* tended to be more prevalent in female sepsis patients than in males, and *Chimonocalamus* tended to be more abundant in female trauma patients compared to males. A higher level of *Rhodotorula* was observed in female subjects across all groups (Fig. 2c–e). Although no taxa were identified as significantly differing by gender in ANOVA, *Rhodotorula* and *Chimonocalamus* were the most affected taxa by gender, regardless of the group, among all taxa (Fig. 2f). We further trained and executed a machine-learning model to predict and capture the primary distinctions between sepsis or trauma patients and healthy subjects, aiming to understand the abnormalities of their mycobiome. The control group demonstrated the highest prediction accuracy (AUC = 0.92); while, the sepsis and trauma groups exhibited relatively lower accuracy (AUC = 0.74 and 0.75, respectively) (Fig. 2h). Within the sepsis group, samples that were incorrectly predicted were more likely to be categorized as belonging to the trauma group rather than the control group, and the same pattern was observed within the trauma group (Fig. 2g). This may be attributed to the similar microbial profiles observed in both sepsis and trauma patients. *Candida* was consistently identified as the most important feature for prediction, followed by *Curvularia* and *Penicillium*, and these taxa were most abundant in sepsis, trauma, and control groups, respectively (Fig. 2i).

In each analysis, *Candida* reproducibly proved to be the most significant and distinctive genus for distinguishing sepsis and trauma patients from healthy adults. We investigated whether the *Candida* profile alone could accurately characterize the groups and be exclusively utilized as a group prediction factor. Overall, not only the abundance but also its detection rate in sepsis and trauma groups was significantly higher than in the healthy control group (Fig. 2j). Although the average abundance of *Candida* was highest in the sepsis group, the detection rate was slightly lower than in the trauma group, where all patients had *Candida* carriage. All the ASVs identified as *Candida* were successfully assigned to the species level, resulting in six species. *Candida* species profiles exhibited substantial differences between the healthy control group versus sepsis and trauma patients; while, sepsis and trauma patients shared relatively similar profiles. Specifically, *C. orthopsilosis* and *C. sake* were only identified in the healthy control group and represented a significant portion of the *Candida* flora in this group. In contrast, *C. dubliniensis* and *C. parapsilosis*, which were major species in the sepsis and trauma groups, were either not detected or barely detected with very low abundance. *C. albicans* and *C. tropicalis* were the most abundant and highly detected species in all groups, although the sepsis and trauma groups had relatively higher abundance and detection rates compared to the control group (Fig. 2k). When examining the PCoA analysis based on *Candida* species profiles, the healthy control group exhibited a distinct profile and was significantly separated from the sepsis and trauma groups. Intriguingly, the overall profile within each group was nearly identical to the group structures generated with all ASVs, indicating that ASVs assigned to *Candida* species were the major features determining the overall group structures and driving the differences between the groups (Fig. 2l). Furthermore, the prediction accuracy of the model trained solely with *Candida* species showed analogous patterns to the prediction model trained with all microbiome data (Fig. 2m, n). *C. tropicalis* and *C. albicans* were consistently identified as the most important species for determining and predicting the groups (Fig. 2o).

### Mycobiome dysbiosis signatures correlate with perturbed bacteriome-metabolome micro-ecological niches

**Fig. 3:**
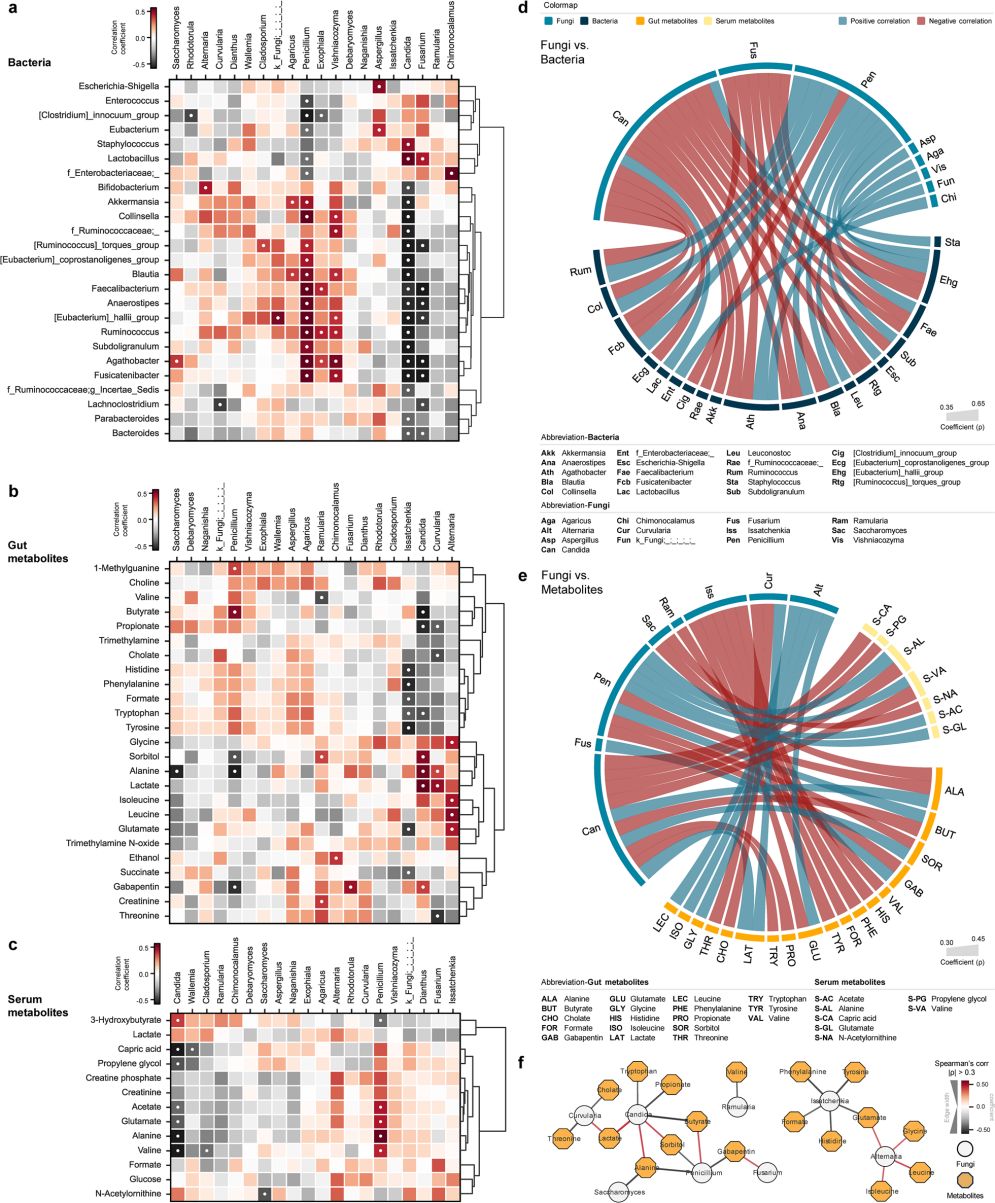
Mycobiome dysbiosis signatures correlate with perturbed gut bacteriome and gut-plasma metabolome arrays. Correlation arrays between mycobiome and **a** microbiome, **b** gut metabolites, and **c** plasma metabolites calculated with all samples from control, trauma and sepsis cohorts (Statistical significance: ● *p *< 0.05) Significant correlation networks **d** between mycobiome and microbiome (shown *ρ *> 0.35) and **e** between mycobiome and gut and plasma metabolites (shown *ρ *> 0.35). **f** Mycobiome-gut metabolites co-occurrence network. Circular nodes represent Fungi; while, yellow octagon nodes represent gut metabolites. Only significant links are shown here (Spearman’s rank correlation coefficient (*ρ*) > 0.3; Benjamini–Hochberg corrected *p *< 0.05). Red links denote positive correlation and black links indicate negative correlation, with line thickness corresponding to the correlation coefficient value

The gut is a highly complex ecosystem with various forms of symbiosis. Changes in one domain vastly affect other domains and the microbial by-products produced after their metabolism. We have previously observed significant reduction in the gut bacterial diversity in sepsis and trauma patients, accompanied by significant shifts in the composition of the microbiota, including the emergence of specific pathobiome patterns. Additionally, both trauma and sepsis were associated with discernible changes in fecal and plasma metabolites (Additional file 2: Figure S1). Therefore, to understand the ecological niche and fundamental co-regulation of mycobiome communities within the gut, we applied correlational analyses of fungal taxa with gut bacteriome as well as with gut and plasma metabolome arrays (Fig. 3a–e). Bacterial taxa that had at least one significant co-occurring relationship with fungal taxa were divided into two branches based on their hierarchical clustering of correlational relationships. One branch primarily consisted of bacteria associated with gastrointestinal infections, such as the Enterobacteriaceae family, *Escherichia–Shigella*, *Enterococcus*, and *Staphylococcus*. These bacteria tended to positively correlate with fungal taxa found abundant in sepsis and trauma patients. The other branch mostly contained commensal and beneficial taxa, including *Bifidobacterium*, *Akkermansia*, and *Ruminococcus*, which tended to co-occur with fungal genera more abundant in healthy adults (Fig. 3a). Specifically, three major genera, *Candida*, *Fusarium*, and *Penicillium*, exhibited the most significant correlations with commensal bacteria, including *Faecalibacterium*, *Anaerostipes*, and the *[Eubacterium] hallii* group. *Candida* and *Fusarium*, which were increased in sepsis and trauma patients, negatively correlated with these bacteria, while *Penicillium*, more abundant in the healthy control group, showed a strong positive correlation with these bacteria. Additionally, several beneficial bacteria, such as *Akkermansia* and *Blautia*, demonstrated a positive correlation with *Penicillium* and a negative correlation with *Candida*. Meanwhile, we observed a reverse trend with *Lactobacillus*, which was positively correlated with fungal taxa abundant in sepsis and trauma patients (Fig. 3d).

Gut microbial metabolites were also clustered hierarchically based on their co-regulation networks with fungal taxa. One cluster contained short-chain fatty acids (SCFAs), including butyrate and propionate, which were positively correlated with fungal taxa prevalent in healthy adults. Another cluster consisted of by-products of microbial metabolism, including lactate, trimethylamine-N-oxide (TMAO), and ethanol, which were positively correlated with taxa more abundant in sepsis and trauma patients (Fig. 3b). *Candida* showed a strong negative correlation with SCFAs; while, *Penicillium* demonstrated a robust positive correlation with butyrate. These results were also reflected in the network analysis, where *Candida* showed a co-occurrence network with alanine and sorbitol and a mutual exclusive network with butyrate. In contrast, *Penicillium* showed exactly opposite networks with these metabolites (Fig. 3f). *Curvularia* exhibited a similar correlational profile with *Candida*, primarily negatively correlating with essential metabolites associated with gut integrity, such as propionate, cholate, and threonine. While *Issatchenkia* and *Alternaria* exhibited several significant correlations and networks with amino acids, their low prevalence rates (9.4% and 5.7%, respectively) suggest that the significant results may be misleading due to the skewed distribution of data. In blood (plasma) metabolome, three metabolites, including lactate, glutamate, and alanine, were found to be common with gut metabolites. Among these, only lactate displayed a similar correlational pattern with fungi as observed in the gut lactate, although it did not reach statistical significance. Overall, only *Candida* and *Penicillium* demonstrated strong and distinct correlations with plasma metabolites, and these two genera had inverse profiles. *Penicillium* negatively correlated with 3-hydroxybutyrate, the primary ketone bodies produced during ketogenesis, while positively correlating with acetate, glutamate, and alanine, which are involved in gluconeogenesis. *Candida* however showed an inverse relationship with these metabolites (Fig. 3c, e).

## Discussion

The microbiome serves as the first line of defense against gut pathogens, and disturbances in the gut microbiome (dysbiosis) can lead to an increased susceptibility to serious infections. Indeed, previous studies have shown that patients exposed to antibiotic treatment during hospital stay are at a higher risk of subsequent sepsis-related hospitalization, primarily because of gut dysbiosis and pathobiome [32]. While substantial disruptions in the mycobiome are envisaged in septic or post-injury patients during hospitalization, the research in this area remains limited, with studies focusing solely on the bacterial microbiome. Herein, we demonstrate the presence, persistence, and phenotype of the gut mycobiome dysbiosis in septic or post-injury patients after two weeks of hospitalization. The findings reveal the existence of late dysbiosis in critically ill sepsis and trauma patients who do not rapidly recover and remain in the ICU for at least two–three weeks or longer. Notably, the incidence of recidivism and the predisposition to poor long-term outcomes is high in these patients.

Overall, we identified dysbiosis in the mycobiome characterized by reduced diversity (species richness), altered microbial structure, a sharp increase in *Candida*, and a depletion of commensal fungal taxa, including *Penicillium* and *Saccharomyces*, in both sepsis and trauma patients. Given the fact that these fungi are the major genera comprising the mycobiome community with high prevalence, the sepsis and trauma patients may experience a significant disturbance in the fungal ecological niche and are exposed to a high risk of subsequent fungal infections like candidiasis. While no specific studies have reported overall mycobiome changes in sepsis and trauma patients, there is a consensus that critically ill patients in the ICU have an infection-susceptible gut micro-environment [20]. Previous studies have reported that the bacterial community is severely disrupted, and the fungal community, which has mutualistic interactions with the bacterial community, is also altered during the ICU stay. As a consequence, immunomodulatory metabolites and key metabolites, including SCFAs and bile acids (BAs), are depleted or altered [20]. Additionally, the observation of similar pattern in two distinct medical conditions provides evidence of a potential causal link between trauma and the development of sepsis in these patients. Previous studies have reported that severe trauma can induce drastic dysbiosis within a short period and lead to sepsis due to disruptions in the intestinal epithelial barrier [33, 34]. We also observed a difference in microbial diversity changes between the sexes. Male patients demonstrated a drastic decrease in diversity, especially among the sepsis cohorts, whereas female patients' microbial diversity either slightly decreased or barely changed, suggesting that males experienced relatively more severe dysbiosis. This finding aligns with our previous observations regarding bacterial communities, where the female microbiome displayed better resistance to infection or injury [24, 35, 36]. These results might suggest that there is a tendency for severe sepsis to be more prevalent in men, and sex dimorphism in dysbiosis may be one of the crucial reasons for the higher severity and prevalence of sepsis in males [37, 38].

One of the strongest factors driving these dysbiotic conditions is undoubtedly the antibiotics regimen. Antibiotic administration can lead to an overgrowth in *Candida* while disrupting the overall microbial communities, as observed in this study [20, 39, 40]. In total, six *Candida* species were identified, with four species found to be more abundant in sepsis and trauma patients. *C. parapsilosis* and *C. dubliniensis*, exclusively detected in sepsis trauma patients, are not commonly found in the gut, but rather on the hands and in the oral cavity, respectively [41, 42]. Whereas *C. albicans* and *C. tropicalis* were found in all groups but were more prevalent and abundant in sepsis and trauma patients. Both species are taxonomically close and share pathogenic traits [43], making them well-known inducers of candidiasis and often found in ICU patients treated with antibiotics. [20]. Taken together, both exogenous and endogenous *Candida* species readily proliferated within the gut of sepsis and trauma patients because of their disrupted ecological niches and immunocompromised state. Furthermore, studies on patients or neonates in the ICU have suggested that broad-spectrum antibiotics and high exposure to antibiotic therapy may contribute to the incidence of invasive candidiasis [44, 45]. Additionally, the expansion of pathogenic *Candida* species can be an early marker of systemic candidiasis [8]. The mechanistic pathways through which these species dominate the gut during dysbiosis and induce candidiasis may be multi-factorial and multi-dynamic. For instance, the loss of commensal bacteria and concomitant SCFAs reduction can trigger or inhibit the proliferation of *Candida*. Specific commensal bacteria have been shown to inhibit *C. albicans* colonization by activating innate immune effectors and antimicrobial peptides [40]. Peptidoglycan directly affects *C. albicans* growth by activating the adenylyl cyclase Cyr1p, which controls hyphal morphogenesis [46]. Antibiotic treatment can increase the availability of peptidoglycan fragments by facilitating the release of peptidoglycan subunits, leading to *C. albicans* hyphal growth [47]. Our co-regulation analyses revealed a strong negative correlation of *Candida* with various commensals, including gram-positive ones. This suggests that sepsis and trauma patients not only lose inhibitory effects from compromised commensals but also develop a favorable ecological niche for the proliferation of *Candida* due to increased peptidoglycan fragments. SCFAs have inhibitory effect against the growth and development of *Candida* components, including germ tubes, hyphae, and biofilms; accordingly, reduced SCFAs levels have been linked to increased *C. albicans* susceptibility in animal models [39]. Likewise, our findings showed a strong negative correlation of *Candida* levels with SCFAs, indicating the weakening or loss of *Candida*-suppressive effects of SCFAs in sepsis and trauma patients due to depleted population of SCFAs-producers.

While we observed a diminution of *Penicillium* and *Saccharomyces* in sepsis and trauma patients, it may be noted that *Penicillium* spp. are known to produce diverse secondary metabolites, including fatty acids with antibacterial activities [48]. Though there is some argument regarding the viability of *Penicillium* in the gut environment due to growth constraints in certain species [49], it has been identified with high abundance in healthy adults [50, 51] and has demonstrated antimicrobial effects against pathogenic bacteria [52]. Similar to *Penicillium*, *Saccharomyces* has also been found in high proportions in healthy adults and exhibits a strong negative correlation with *Candida* [53, 54]. *Saccharomyces* can also effectively serve as a functional replacement for intestinal bacteria, providing protection against mucosal tissue damage and enhancing the responsiveness of circulating immune cells [55]. Studies have shown that the administration of *S. cerevisiae* in antibiotic-treated mice not only ameliorates dysbiosis by restoring bacterial commensals [56, 57], but also significantly improves mortality and susceptibility to viral infections [55]. Although there are conflicting results regarding the inhibitory effect of *Saccharomyces* on *C. albicans* [58], several studies have demonstrated that the administration of *Saccharomyces* sp. reduces the intestinal colonization of *C. albicans* and the incidence of invasive candidiasis [59, 60].

Importantly, we observed significant correlations of mycobiome with altered metabolomic niches. Among these associations, one important observation was for plasma 3-hydroxybutyrate. As previously mentioned, it is one of the ketone bodies synthesized by the liver during periods of low carbohydrate intake or fasting [61]. Elevated levels of this compound are observed in sepsis and trauma patients, consistent with prior research on sepsis patients [62, 63], indicating that these patients may enter in a ketogenic state due to the scarcity of glucose in their blood and inadequate gluconeogenesis. Critically ill patients frequently experience hypoglycemia [64], likely due to mitochondrial disorders induced by oxidative stress [65, 66]. Additionally, patients in the ICU receiving enteral nutrition often encounter enteral feeding intolerance, primarily because of altered gastrointestinal motility and gastroparesis [67] with its prevalence reaching up to 75% (38.3% on average) [68]. Together, these and our findings suggest that sepsis and trauma patients may exhibit abnormal blood sugar levels and reduced sugar availability in the gut due to their medical conditions. In our correlational analysis, *Candida* displayed a positive association with plasma 3-hydroxybutyrate and gut lactate. Previous studies have indicated that severe hyperglycemia and ketonemia, characterized by high levels of 3-hydroxybutyrate, can lead to a reduction in human antigen-specific T cell proliferation. This reduction makes patients more susceptible to fungal infections, particularly those caused by *C. albicans*, as well as manifested *Candida* sepsis [69, 70]. These results imply that during the ICU stay, patients may be exposed to a higher risk of *Candida* infection because of the prolonged ketonemic state. In addition, *Candida*, especially *C. albicans*, is known for its remarkable metabolic flexibility, allowing it to utilize a variety of nutrients in sugar-limited environments, with lactate being the most readily available alternative carbon source. Intriguingly, *C. albicans* grown on lactate is less detectable by the host immune system and thus are less efficiently phagocytosed by immune cells [71]. This suggests that altered nutrient availability in host niches during sepsis and trauma not only promotes the growth of fungal pathobionts but may also enhance their virulence.

## Conclusions

In summary, our findings for the first time unveil the persistence and signatures of the gut mycobiome dysbiosis shifting toward an infection-susceptible pathobiome state two weeks after sepsis or trauma. Like any study of this nature, our study has limitations. We were able to collect samples at a single timepoint, because of which we could not investigate the trajectory of major fungal taxa over time and the duration of hospitalization. Although there were no differences among groups in terms of ethnicity, most of the participants were Caucasian. Additionally, we failed to control for other factors that might possibly affect the mycobiome, including the type of enteral nutrition and anesthesia [72, 73]. Nevertheless, the study was adequately powered for each group, revealing the persistence of mycobiome dysbiosis in both sepsis and trauma settings, even up to two weeks post-sepsis and trauma. The tenacity of these perturbations in patients with delayed or complicated clinical trajectories strongly suggests that targeting this dysbiosis could be a viable focus for therapeutic regimens aimed at reducing the risk of subsequent fungal infections. Such regimens might employ anti-*Candida* microbiome rehabilitation strategies such as *S. cerevisiae* administration or pro/pre/postbiotic pharmacological activation of the innate immune defense system against colonization by opportunistic pathogenic fungi [40]. The findings call for and should facilitate future studies to explore novel approaches to minimize the incidence of fungal infection and develop therapeutic interventions aimed at preventing dysbiosis and restoring gut micro-ecological homeostasis for improved health outcomes and quality-of-life following recovery from hospitalization and critical illness.

### Supplementary Information

**Additional file 1: Table S1.** Inclusion and exclusion criteria. **Table S2.** Patient characteristics.

**Additional file 2: Fig. S1.** Overall profiles of bacterial community and gut and serum metabolites of cohorts. **a** Bacterial alpha-diversity comparing control, sepsis and trauma cohorts represented by Faith’s phylogenetic diversity (PD). **b** Principal coordinate analysis (PCoA) plots showing differences in beta-diversity (unweighted and weighted UniFrac distance) between control, sepsis and trauma cohorts with PERMANOVA *p* values between cohorts. Relative abundance of **c** major 15 genus, 10 gut metabolites, and 5 plasma metabolites. Data are presented as mean ± SE. *p < 0.05, **p < 0.01, ***p < 0.001.

## Data Availability

The datasets generated and/or analyzed during the current study are available in the National Center for Biotechnology Information (NCBI)’s Sequence Read Archive (SRA) (www.ncbi.nlm.nih.gov/sra; Bioproject #PRJNA1030809).

## Abbreviations

ASV: Amplicon sequence variant
BA: Bile acid
ALDEx: ANOVA-like differential expression
CCI: Chronic critical illness
GCS: Glasgow coma scale
ICU: Intensive care unit
IRB: Institutional review board
ITS: Internal transcribed spacer
PCoA: Principal coordinate analysis
PICS: Prolonged inflammation, immunosuppression, and catabolism
QIIME: Quantitative insights into microbial ecology
SCFA: Short chain fatty acid
STROBE: Strengthening the reporting of observational studies in epidemiology
TBI: Traumatic brain injury
TBSA: Total body surface area
TMAO: Trimethylamine-N-oxide
