# Supplementary material for: Gut mycobiome dysbiosis after sepsis and trauma
Source: Crit Care. 2024 Jan 11;28:18. doi: 10.1186/s13054-023-04780-4 (PMC10785534; doi:10.1186/s13054-023-04780-4)
Supplement: Supplementary file 1 — Additional file 1: Table S1. Inclusion and exclusion criteria. Table S2. Patient characteristics. [file 13054_2023_4780_MOESM1_ESM.docx]

**Table S1**. Inclusion and exclusion criteria

| Subject group | Healthy subjects | Sepsis patients | Trauma patients |
| --- | --- | --- | --- |
| Inclusion criteria | - over 18 years of age - capable of providing informed consent | | |
|  | - | - meet the Sepsis-3 criteria - have an ICU stay of at least seven days following a sepsis diagnosis - receive oral or enteral nutrition were included | - have experienced blunt or penetrating trauma with an Injury Severity Score of  15 or higher - have hemorrhagic shock on admission, defined as a systolic blood pressure of  90 mmHg or lower, a mean arterial pressure of 60 mmHg or lower, a base deficit of 5 mEq or higher, a lactic acid level of 2 mmol/L or higher - have received active blood transfusion within 6 hours of arrival |
| Exclusion criteria | - pregnancy | | |
|  | - currently or chronically using steroids or antibiotics | - with severe traumatic brain injury (TBI) presenting a Glasgow Coma Scale (GCS) score of 3 upon admission - have burns covering more than 20% of their total body surface area (TBSA) - who are incarcerated or institutionalized | |
|  |  | - have refractory shock - with an uncontrollable source of sepsis or an irreversible disease state - individuals not expected to survive for more than 12 hours, organ transplant recipients - have immunosuppressive agents | - with end-stage renal disease, pre-existing hematological diseases, a history of bone marrow transplantation - individuals not expected to survive for more than 48 hours |

**Table S2**. Patient characteristics

| Subject group | Healthy subjects | Sepsis patients | Trauma patients | p-value |
| --- | --- | --- | --- | --- |
| Number of subjects | 19 | 18 | 18 | - |
| Number of male subjects, n (%) | 11 (58%) | 10 (56%) | 11 (61%) | 0.94 |
| Race | | | | |
| White, n (%) | 17 (90%) | 17 (94%) | 15 (83%) | 0.56 |
| Black, n (%) | 1 (5%) | 1 (6%) | 3 (17%) | 0.39 |
| Asian, n (%) | 1 (5%) | 0 (0%) | 0 (0%) | 0.38 |
| Age | 54.7 ± 10.5 | 60.1 ± 15.8 | 55.0 ± 16.9 | 0.48 |
| Hospitalization | | | | |
| SOFA score | - | 6.0 ± 2.5 | - | - |
| APACHE II score |  | 16.0 ± 5.6 | - | - |
| Injury Severity score (ISS) |  | - | 26.0 ± 9.7 | - |
| Blunt trauma, n (%) |  | - | 18 (100%) | - |
| Number of surgical procedures |  | 4.3 ± 3.8 | 3.4 ± 2.2 | 0.39 |
| Number of Antibiotics administrated |  | 6.4 ± 2.1 | 4.6 ± 3.0 | **0.04** |
| Total days of antibiotics administration |  | 22.3 ± 15.2 | 10.1 ± 8.3 | **0.006** |
| ICU length of stay (days) |  | 19.0 ± 10.9 | 12.4 ± 7.2 | **0.04** |
| Hospital length of stay (days) |  | 30.6 ± 19.5 | 19.7 ± 7.0 | **0.04** |
| Hospital day of stool collection |  | 18.1 ± 8.1 | 13.7 ± 5.3 | 0.07 |
| Hospital day of plasma collection |  | 17.6 ± 8.1 | 13.0 ± 6.7 | 0.08 |

* SOFA: sequential organ failure assessment; APACHE: acute physiology, age and chronic health evaluation; ICU: intensive care unit
