# Supplementary figures and images for: Gut mycobiome dysbiosis after sepsis and trauma
Source: Crit Care. 2024 Jan 11;28:18. doi: 10.1186/s13054-023-04780-4 (PMC10785534; doi:10.1186/s13054-023-04780-4)

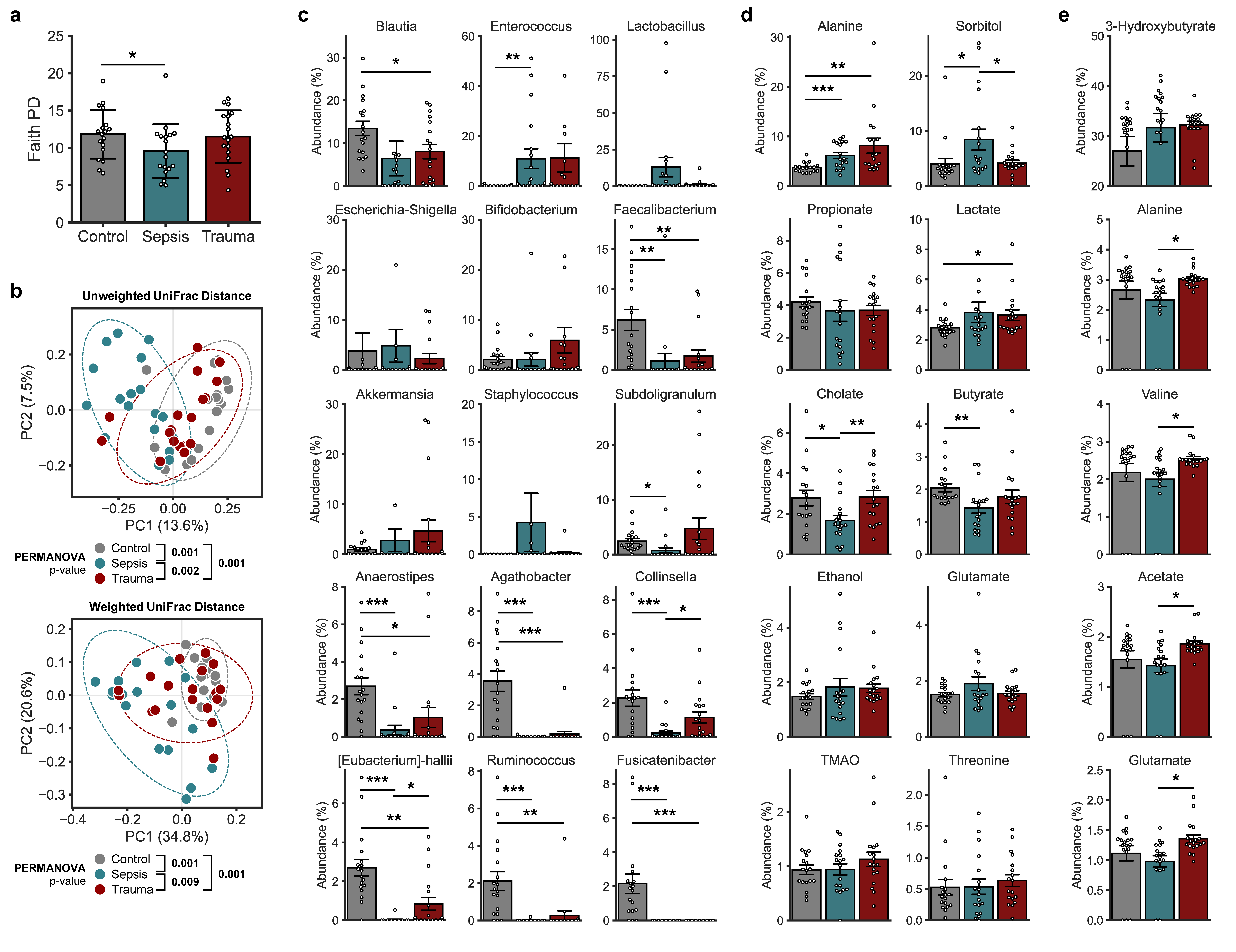

Supplement: Supplementary file 2 — Additional file 2: Fig. S1. Overall profiles of bacterial community and gut and serum metabolites of cohorts. a Bacterial alpha-diversity comparing control, sepsis and trauma cohorts represented by Faith’s phylogenetic diversity (PD). b Principal coordinate analysis (PCoA) plots showing differences in beta-diversity (unweighted and weighted UniFrac distance) between control, sepsis and trauma cohorts with PERMANOVA p values between cohorts. Relative abundance of c major 15 genus, 10 gut metabolites, and 5 plasma metabolites. Data are presented as mean ± SE. *p < 0.05, **p < 0.01, ***p < 0.001. [file 13054_2023_4780_MOESM2_ESM.tiff]
